# Strategies for Intravenous Fluid Resuscitation in Trauma Patients

**DOI:** 10.1007/s00268-016-3865-7

**Published:** 2017-01-05

**Authors:** Robert Wise, Michael Faurie, Manu L. N. G. Malbrain, Eric Hodgson

**Affiliations:** 1 Pietermaritzburg Metropolitan Department of Anaesthetics, Critical Care and Pain Management, Pietermaritzburg, South Africa; 2 Head Clinical Unit, Critical Care, Edendale Hospital, Pietermaritzburg, South Africa; 3 Discipline of Anaesthesia and Critical Care, School of Clinical Medicine, College of Health Sciences, Nelson R Mandela School of Medicine, University of KwaZulu-Natal, Durban, South Africa; 4 Trauma and Critical Care Fellow, Inkosi Albert Luthuli Central Hospital, Durban, South Africa; 5 Ziekenhuis Netwerk Antwerpen, ZNA Stuivenberg, Lange Beeldekensstraat 267, 2060 Antwerpen 6, Belgium; 6 Inkosi Albert Luthuli Central Hospital, Durban, South Africa

## Abstract

Intravenous fluid management of trauma patients is fraught with complex decisions that are often complicated by coagulopathy and blood loss. This review discusses the fluid management in trauma patients from the perspective of the developing world. In addition, the article describes an approach to specific circumstances in trauma fluid decision-making and provides recommendations for the resource-limited environment.

## Initial resuscitation fluid management

### Background

Fluid resuscitation of trauma patients has been an ongoing challenge, constantly reviewed and debated, resulting in recommendations changing for the use of crystalloids/colloids/packed red blood cells/warm fresh whole blood and clotting factors. Other challenges, such as limited resources, impact the practitioners’ choice of fluid—the best fluid available does not always equate to the best fluid for the patient, especially where long transfers and no blood availability are concerned. These decisions and management strategies appear relevant for further discussion and research, as this fluid resuscitation attempts to provide adequate organ perfusion and oxygen delivery in a system compromised by the physiological consequences of injury. Several questions have arisen from this topic: Which fluid is best, how much should be given, and do specific injuries call for different strategies (for example, penetrating vs. blunt trauma)? Achieving balance in the resuscitation period is challenging, particularly the volume administered. More fluid is not always better, in fact, quite the contrary [1–3]. Much of the literature on fluid resuscitation focuses on critically ill patients with sepsis, or elective perioperative patients [4–7]. Small cohorts of trauma patients can be found in the larger studies, but it should be remembered that most of these data include patients from the ICU setting [4, 5]. Extrapolation of data to the initial resuscitation phase of the trauma patient is not possible [8, 9]. This article emphasizes different types of fluid available, when they should be used, and recommendations on how to tailor fluid resuscitation through monitoring techniques. The goals of improving physiology, restoring or maintaining normothermia and minimizing coagulopathy should be considered paramount throughout the discussion.

### Penetrating versus blunt injury versus head injuries

There are three distinct groups of trauma patients, but often there is an overlap. Most commonly encountered is the combination of blunt trauma and head injury associated with motor vehicle collisions. While the general Advanced Trauma Life Support (ATLS) management approach to the three groups remains similar, fluid therapy strategies differ. The literature suggests patients with penetrating injuries, particularly to the thoracoabdominal region, have better outcomes with restrictive clear fluid resuscitation policies, permitting a systolic blood pressure (BP) between 60 and 70 mmHg until the patient can be taken to the operating theater [10]. Once hemorrhage has been controlled in theater and blood products are available, higher blood pressure values may be targeted. There have been no large trials comparing restrictive and liberal fluid strategies in the context of blunt injury. However, a restrictive policy is acceptable with slower infusions favored over rapid boluses [10]. A slightly higher systolic blood pressure of 80–90 mmHg is permitted, again, until control in theater is achieved and blood products are available. This restrictive policy is thought to minimize intra-abdominal bleeding while maintaining adequate organ perfusion and reducing the risk of intra-abdominal hypertension and complications mentioned previously. It should be remembered, however, that clinical scenarios are often complicated, and blood pressure goals should be individualized according to patient physiology, comorbidities and physiological compensation to shock during the time of resuscitation.

The exception to the guidelines above is the polytrauma patient (blunt or penetrating) with traumatic brain injury (TBI). In order to preserve adequate cerebral perfusion pressure and prevent secondary brain injury, one needs to target a mean arterial pressure (MAP) of greater than 80 mmHg (a cerebral perfusion pressure of approximately 60 mmHg) [10].

### Clear fluid resuscitation

The ongoing debate as to which group of fluid (synthetic colloid or crystalloid solutions [3]) is best to use in the resuscitation phase of trauma patients remains unanswered with large studies showing little, if any benefit of hydroxyethyl starch 130/0.4 [11] over the traditionally used crystalloids. The CRISTAL trial did identify a potential mortality benefit in a heterogeneous hypovolemic patient cohort resuscitated with a variety of colloid solutions compared to crystalloid solutions. However, several limitations identified by the authors limits applicability: the lack of renal injury and potential 90-day outcome benefit, deserve further research [7]. When reviewing the available literature, in several trials recruitment and consent requirements resulted in the comparison of fluids commencing after the initial resuscitation phase, resulting in interpretation difficulties of outcome benefit in trauma patients [4, 5, 7, 12]. These studies do, however, demonstrate a trend toward less synthetic colloid fluid required to achieve hemodynamic goals compared to crystalloids with a ratio (volume of colloid to crystalloid that results in similar physiological effects) varying between 1:1.1 and 1:1.6 (colloids:crystalloids) [4, 5, 7, 12]. This ratio is smaller than previously thought (ATLS teaches a ratio of 1:3), and significance in subgroups of patients is yet to be determined. Concerns still exist about the adverse effects of hydroxyethyl starch 130/0.4 on renal function and coagulopathy although crystalloid fluids are not without complications [7]. Further studies need to be done comparing these crystalloids and colloids in the initial resuscitation phase of trauma patients. A further concern is the chloride load administered in these fluids and the potential contribution toward acidosis and renal injury [13]. The resultant hyperchloremic metabolic acidosis may have negative consequences. The meta-analysis by Krajewski et al. [13] showed a significant association between high chloride content fluids and acute kidney injury, blood transfusion volume and mechanical ventilation time. Mortality was unaffected in this population of perioperative patients. Despite this, 0.9% saline remains widely used as a resuscitation fluid and remains the fluid of choice for patients with brain injury, hyponatremia and metabolic alkalosis. Balanced salt solutions (solutions with a physiological pH and isotonic electrolyte concentration), being more physiological in nature, are being used more frequently, showing a trend toward less harm than 0.9% sodium chloride—whether in isolation or as a medium carrying a colloid [14]. Balanced salt solutions closely resemble human plasma and thus have a lower sodium and chloride content than 0.9% saline with the addition of a buffer such as acetate or lactate. These fluids (e.g., Ringer’s lactate, Hartmann’s solution) have minimal effects on pH but are hypotonic, so can exacerbate edema, particularly cerebral edema in the injured brain. In addition, when using Ringer’s lactate solution, consideration should be given to the potential interaction between citrate found in stored blood and bicarbonate, explaining why 0.9% saline is still a commonly used resuscitation fluid in trauma patients, despite the high chloride load [15]. The concerns regarding the inflammatory effects from Ringers lactate infusions, demonstrated in animal models, have not been demonstrated to influence outcomes in human studies. Of greater concern are the negative consequences of hyperchloremic metabolic acidosis. There are no large randomized control trials demonstrating a mortality benefit for 0.9% saline over balanced solutions. Currently, saline is preferred in brain-injured patients and balanced solutions are preferred in patients who are already acidotic. Although only in an porcine model, resuscitation after severe hemorrhage with 0.9% saline was inferior to ringers lactate due to vasodilatory effect, and risks of metabolic acidosis and hyperkalemia [16]. In elective neurosurgical patients, lactated ringers also proved better than 0.9% saline in terms of electrolyte management (particularly sodium and chloride) and acid–base balance [17].

In the resource-limited environment, the use of cheaper crystalloid solutions is still recommended due to lack of data showing significant outcome benefits of more expensive synthetic colloids. A selection of different crystalloid solutions is often not available in resource-limited settings, making the available solution the only and best choice. In a Cochrane review, the use of hypertonic saline for the resuscitation of trauma victims has failed to show any benefit over isotonic or near-isotonic crystalloids and two adequately powered trials investigating mortality as an endpoint were halted early due to futility. Controversy, however, still continues fueled by animal studies demonstrating benefits that have not been reflected as outcome benefits in human studies [18]. Heterogeneous populations and methodological differences between studies make the interpretation of evidence difficult. Hypertonic saline may have a role when used in the head-injured patient as a bridge to neurosurgery [19].

Several trials show either no benefit, or in some cases worse outcomes, with albumin thus making this solution not recommended in the resuscitation of trauma patients [20–22].

The physiological impact of the volume of fluid infused may be as, or even more important than the type selected [2, 23–26]. Excessive fluid results in a dilutional coagulopathy and diffuse tissue edema. This negatively impacts organ function at both a macroscopic and cellular level by increasing the distance over which electrolytes, elements and oxygen have to move [3]. The consequence is worsening renal, hepatic and cardiac function as well as increasing volume of extra vascular lung water that worsens ventilation–perfusion mismatch. Abdominal hypertension/compartment syndrome may progress to a polycompartment syndrome [23, 26].

Therefore, until such time as blood and blood products are available, clear fluid resuscitation should be limited to only that which is necessary to maintain adequate organ perfusion. Several factors influence decisions at this point of the resuscitation. Trauma units with easy access to on-site blood and blood products should commence resuscitation of patients with massive blood loss with these products from the start. In environments where blood products are limited, the authors suggest judicious use of clear fluids to sustain organ perfusion while avoiding the negative effects of excess fluid. Response to fluid administration and determining the need for further fluid administration is discussed in the next section.

As defined by the Advanced Trauma Life Support course, classification of patients into those that respond to initial fluid resuscitation versus those that only transiently respond or do not respond at all is important. The response to intravenous fluid resuscitation is assessed using physiological markers of improvement such as blood pressure, heart rate, decreasing lactate and normalizing base deficit with adequate control of bleeding. Responders are considered those that demonstrate these physiological improvements, whereas transient responders show an initial improvement followed by further physiological deterioration. Non-responders are those that show continued physiological deterioration despite initial fluid resuscitation. The distinction requires vigilance and repeated clinical assessments to identify those patients with re-bleeding, or ongoing bleeding, and initiation of blood product resuscitation together with surgical intervention. What may be regarded as acceptable physiological parameters will vary depending on many factors including the age, underlying medication and comorbidities of the patient.

**Fig. 1:**
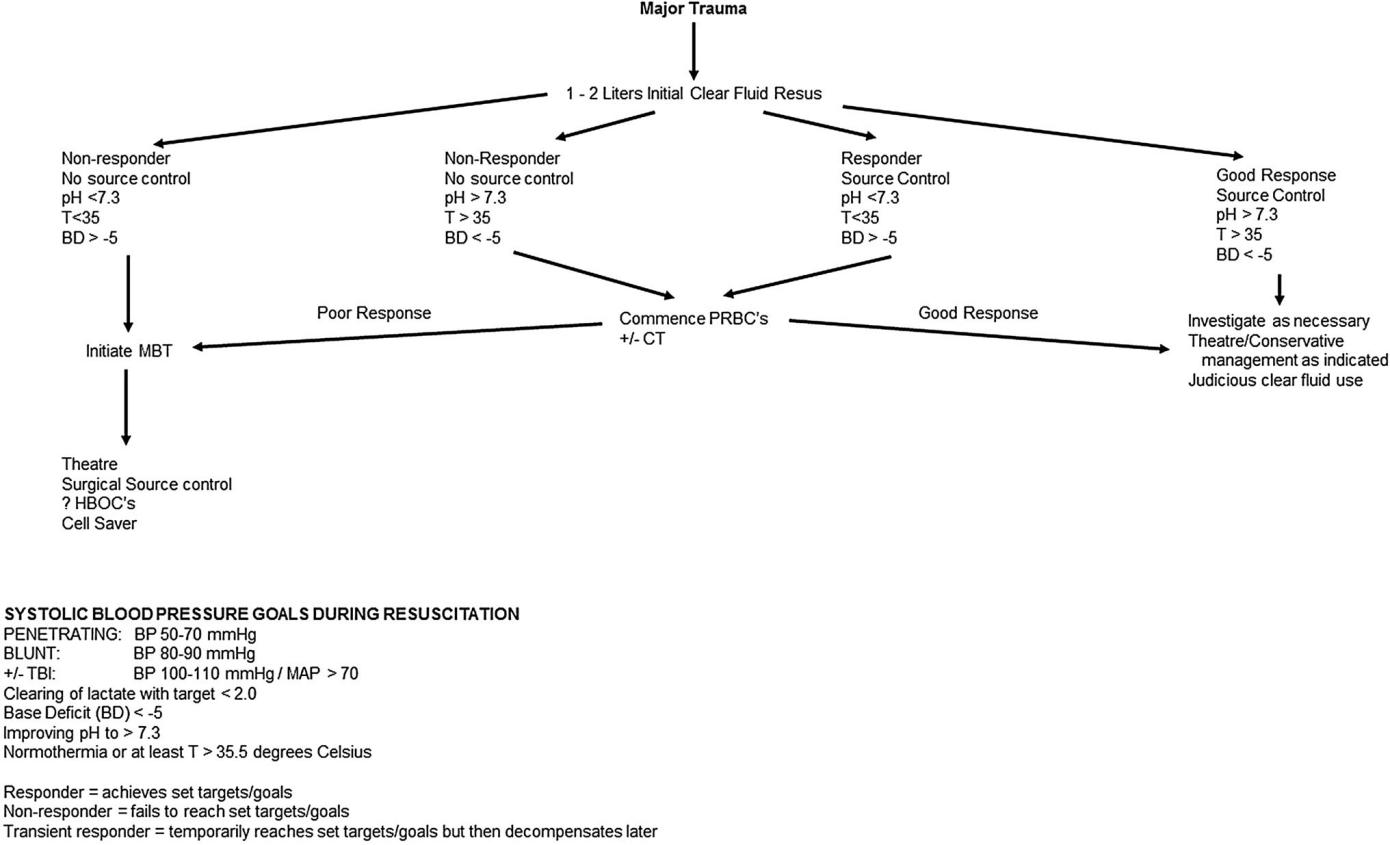
Flow diagram of initial fluid resuscitation of trauma patients

See Fig. 1 for a guide to initial fluid management in trauma patients.

### Blood and blood products

The goal of resuscitation is to achieve adequate tissue perfusion and oxygenation while correcting any coagulopathy. Packed red blood cells, and to some extent hemoglobin-based oxygen carriers (HBOCs), help to achieve the former while component therapy attempts to deal with the coagulopathy. Whole blood may achieve both objectives [27]. Currently, there is no consensus definition for a massive blood transfusion [28]; however, recommendations for the concept of massive transfusions suggest plasma:platelets:red blood cells in a ratio of 1:1:1 or 1:1:2 [29]. This objective is seldom achieved due to limited access and supply of blood products in developing countries, where a 1:1:2 ratio is more easily achieved. An alternative to the use of this ratio is the use of warm fresh whole blood that has a higher hematocrit, more platelets and higher percentage of functional clotting factors per unit volume when compared to component therapy [27]. The concerns about its use, however, include a slightly higher sepsis rate, possible increased risk of acute kidney injury, as well as the risk of transfusion-related immunomodulation (TRIM), although this is minimized by the use of leukodepletion filters [27]. Several trials exist comparing the use of component therapy [30] to warm fresh whole blood, but many did not make this direct comparison, but rather a combination of warm fresh whole blood with packed red blood cells and plasma, making the comparison difficult. Both 24-h and 30-day survival were higher in the warm fresh whole blood/red blood cell/plasma group compared with the component therapy only group, but further trials comparing these groups are necessary [27]. Whole blood also causes less dilutional effect and offers a higher concentration of fibrinogen than component therapy. The current preference for massive transfusion is trending toward the use of whole blood; however, if this is not readily available, as in the resource constrained environments, component therapy with a 1:1:1 ratio should be utilized [27–29, 31, 32].

### Hemoglobin solutions

Modified hemoglobin solutions are not substitutes for blood as they do not possess the metabolic functions of erythrocytes. They act purely as oxygen carriers [33]. Large-scale investigation and experimentation in this field has occurred, with uses not only limited to trauma patients but also general surgical patients, oncology patients and Jehovah’s Witnesses suffering from severe anemia resulting from various reasons [33]. The only product that was registered for use, in South Africa and later Russia, was Hemopure^(R)^ (HbO2 Therapeutics LLC, South Africa), used in isolation as well as in combination with blood products, or as a bridge to blood transfusion [34, 35]. Hemoglobin solutions do not only help oxygen transportation, but also enhance the release of oxygen from native hemoglobin at tissue level with some of them having a positive inotropic effect that may be useful in shocked trauma patients. This positive inotropy is related to the rate of administration and if given slowly is negligible [33, 34]. Serious adverse events (SAEs) were rare with the most serious being fluid overload. A recent review of the literature highlights several flaws in Natanson’s previous meta-analysis suggesting that although a small minority of HBOCs have had serious adverse events (myocardial ischemia, cerebrovascular accidents), this cannot be extrapolated as a class effect due to the vast differences among HBOCs with respect to their structure, hemoglobin concentration and nitric oxide scavenging effects. In light of this, there is renewed interest in the use of HBOCs, especially the Hemopure^(R)^ (HbO2 Therapeutics LLC, South Africa) compound, which to date has had the most success with fewest serious adverse events [36]. Further studies are required before these therapies become widely used.

### Monitoring coagulopathy

Trauma-induced coagulopathy [37] is a relatively new concept, and the pathophysiology is still not completely understood. Traditionally, tests such as prothrombin/international normalized ratio [38] and partial thromboplastin time (PTT) were previously used to make this diagnosis. While these tests form the mainstay of trauma-induced coagulopathy testing in many centers, they only focus on the initial part of clot formation and not on the evolution of the clot or clot lysis. D-dimer and fibrinogen levels are used as surrogate markers of fibrinolysis and clotting factor consumption, respectively, but again these are non-specific when assessing coagulation in the injured patient [71]. Diagnosis of trauma-induced coagulopathy using these older assays is defined as: PT > 18 s, INR > 1.5, PTT > 60 s or any of these values at a threshold of 1.5 times their reference value.

**Fig. 2:**
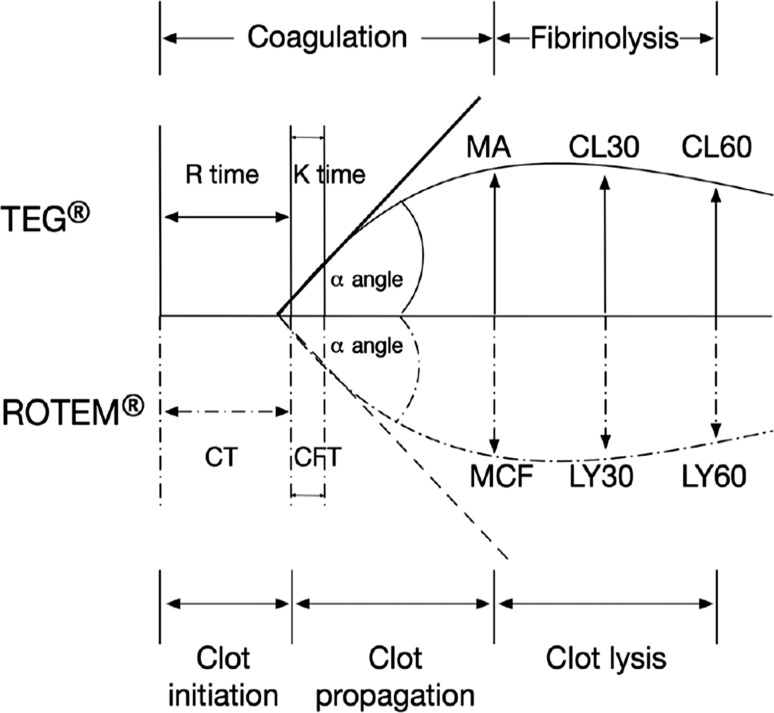
Thromboelastometry—differences in measurement between ROTEM and thromboelastography [42]

Monitoring coagulation in trauma-induced coagulopathy has been made easier with the use of point-of-care testing, namely viscoelastic assays. Recent evidence shows that it improves survival in patients requiring massive blood transfusions compared to those monitored by more traditional assays as described above [39]. While challenges may exist in introducing point-of-care testing into resource-limited settings, these systems would be ideal as they reduce dependency on traditional laboratory testing and allow for real-time feedback and goal-directed blood product use. Viscoelastic assays guide component replacement instead of resuscitating without reproducible biological guidance [40]. By initially assessing patients’ thrombotic deficiencies and continuously re-assessing them following resuscitation with component therapy/whole blood, thromboelastometry guides resuscitation and potentially minimizes the use of allogeneic blood products resulting in reduced risk of transfusion-related side effects and minimizing costs [41]. Table 1 offers a guide to thromboelastometry interpretation and appropriate actions, but a more detailed description and management based on viscoelastic assays is beyond the scope of this review (Fig. 2).

**Table 1:** Thromboelastometry interpretation and action guide

Laboratory value	Interpretation	Blood product transfusion
*R* time <4 min	Enzymatic hypercoagulability	Do not treat if bleeding
*R* time >11 min	Low clotting factors	FDP/FFP’s and RBC’s
Alpha angle >45 degrees	Low fibrinogen levels	Cryoprecipitate/fibrinogen/platelets
MA <54 mm	Low platelet function	Platelets/cryoprecipitate/fibrinogen
MA >73 mm	Platelet hypercoagulability	Do not treat if bleeding
LY30 >3%	Primary fibrinolysis	Tranexamic acid 1 g IV over 10 min then 1 g/250 ml NS over 8 h
CI <1.0		

*FDP* freeze-dried plasma, *FFP* fresh-frozen plasma, *RBC* red blood cell

Recommendations for current best practices:

1. Try and identify risk factors and priorities in trauma patients early (i.e., traumatic brain injury, penetrating injury, ongoing blood losses, compartment syndromes).
2. Consider early administration of blood products in a ratio of 1:1:1 or 1:1:2 if available.
3. In the absence of blood products, use clear fluid resuscitation. Preferably, a balanced salt solution should be used (such as Ringer’s lactate or PlasmaLyte); however, care should be taken not to mix this fluid with blood transfusions.
4. When using clear fluids in resuscitation, vigilance is required to provide only that fluid which is necessary to maintain perfusion. Excessive clear fluid administration has negative consequences such as dilution of coagulation factors, tissue edema, hyperchloremic metabolic acidosis and organ dysfunction.
5. Point-of-care testing should be used whenever possible to guide component therapy replacement to correct coagulopathy.
6. In rural LMIC or long-distance transfer situations with no access to blood, a synthetic colloid may be of benefit in reducing subsequent edema and bowel anastomotic breakdown.

## Post-resuscitation fluid management

### Background

The consequences of under- or over-resuscitation with intravenous fluids are both detrimental [43, 44]. Hypovolemia resulting in adrenergic stimulated vasoconstriction, hypervolemia and fluid overload, massive intravenous fluid administration causing large sodium loads, dilution of coagulation factors, and rapid administration of cold fluids all result in damage to the endothelial layer and glycocalyx, impairment of microcirculatory function and inappropriate fluid shifts. The decision when to transition from an initial resuscitation phase to a post-resuscitation period is critical to a successful outcome.

Following the initial resuscitation phase, physiological targets may change despite the overall objective of adequate tissue perfusion remaining the primary goal. The post-resuscitation period may be considered after:

- Hemostasis and correction of coagulopathy (ongoing blood product replacement no longer required) [45].
- Evidence of improving microcirculatory flow (for example, improving lactate and blood gas parameters) [45].
- Hemodynamic stability (systolic blood pressure >100 mmHg with a mean arterial blood pressure of >65 mmHg in most cases; no longer need for inotropic or vasopressor support; an improving pulse rate in the presence of appropriate analgesia).

At this stage, most patients will no longer be responsive to rapid fluid administration, with normalization of markers for volume status (barometric or volumetric preload indicators) and markers for fluid responsiveness (pulse pressure variation (PPV), stroke volume variation (SVV), passive leg raise (PLR) test) [46].

Importantly, in certain circumstances where patients remain fluid responsive, curtailment of ongoing fluid resuscitation may be necessary if the tolerance to further intravenous fluid is deemed detrimental to physiological processes (for example, abrupt increase in extravascular lung water; worsening intra-abdominal pressure or abdominal compartment syndrome; difficult ventilation) [44]. Alternative strategies should be considered including inotropic support guided by cardiac output monitoring, alternative fluid strategies, and planning for hemodialysis with net ultrafiltration.

The clinician should be aware of the different phases within fluid management according to the ROSE concept. After the initial resuscitation (*R*) phase comes organ support (*O*) and stabilization (*S*), and finally evacuation (*E*) of excess fluids may be needed in some patients (Table 2) [47].

**Table 2:** ROSE concept (adapted from Malbrain et al. with permission) [40]

Resuscitation phase (*R*)
Salvage or rescue treatment with fluids administered quickly as a bolus (4 mL/kg over 10–15 min)
The goal is early adequate goal-directed fluid management (EAFM), fluid balance must be positive, and the suggested resuscitation targets are: MAP > 65 mm Hg, CI > 2.5 L/min/m^2^, PPV < 12%, LVEDAI > 8 cm/m^2^
Optimization phase (*O*)
Occurs within hours
Ischemia and reperfusion
Degree of positive fluid balance may be a marker of severity in this phase
Risk of polycompartment syndrome
Unstable, compensated shock state requiring titrating of fluids to cardiac output
Targets: MAP > 65 mm Hg, CI > 2.5 L/min/m^2^, PPV < 14%, LVEDAI 8−12 cm/m^2^, IAP (<15 mm Hg) are monitored, and APP (>55 mm Hg) is calculated. Preload optimized with GEDVI 640—800 mL/m^2^
Stabilization phase (*S*)
Evolves over days
Fluid therapy only for normal maintenance and replacement
Absence of shock or threat of shock
Monitor daily body weight, fluid balance and organ function
Targets: neutral or negative fluid balance; EVLWI < 10−12 mL/kg PBW, PVPI < 2.5, IAP < 15 mm Hg, APP > 55 mm Hg, COP > 16−18 mm Hg and CLI < 60
Evacuation phase (*E*)
Patients who do not transition from the “ebb” phase of shock to the “flow” phase after the “second hit” develop global increased permeability syndrome (GIPS)
Fluid overload causes end-organ dysfunction
Requires late goal-directed fluid removal (“de-resuscitation”) to achieve negative fluid balance
Need to avoid over-enthusiastic fluid removal resulting in hypovolemia

These are broad guidelines, and exceptions do exist. Urine output has not been included in our recommendations due to the many influencing factors, although it may be one of the only parameters to measure, particularly in resource-limited settings. The renal response to hypovolemia is multifactorial and depends on a combination of renal blood flow, renal perfusion pressure and plasma oncotic pressure. The type of resuscitation fluid is important as it may influence oncotic pressure. In addition, the neurohormonal control of renal function may cloud the clinical picture and appropriate retention of water may be interpreted as renal dysfunction when this may be an appropriate physiological fluid conservation mechanism. As outlined by Peeters et al., previous studies have not found a correlation between urine output and invasively derived physiological variables. Also, several studies have pointed to the inaccuracy of using urine output as a resuscitation target and its limitation in identifying fluid responders. This situation is complicated further in trauma patients in the presence of increased intra-abdominal pressure. The limitations of using urine output should be understood by attending physicians.

Fluids should be treated as drugs: Not only the type of fluid is important, but also the dose, the administration speed, the duration and de-escalation [48]. There are only three indications for giving fluids: resuscitation, replacement or maintenance [49].

### Maintenance fluid

In providing maintenance fluids, care should be taken to avoid causing tissue edema. This requires limitation of crystalloid administration, which can only really be achieved in the post-resuscitation period. Crystalloid fluid administration is not without hazards. Excessive crystalloid administration is associated with edema of skin, abdominal organs (leading to abdominal compartment syndrome), kidneys (leading to renal compartment syndrome, contributing to acute renal failure) and heart (leading to myocardial dysfunction) [44]. The ideal concept would be to use a strategy where the fluid stays intravascular and expands this compartment for longer. However, the design of the recent large studies involving HES showed that ongoing use of these fluids in critically ill patients, beyond initial resuscitation (even in the trauma subgroup analysis), was without benefit and may increase the need for renal replacement therapy [47].

During the post-resuscitation phase, crystalloids are not only required for fluid supplementation, but also as vehicles for administration of medication, including antibiotics, sedation and inotropes/vasopressors. The fluid required for the administration of these solutions together with those required for nutrition should as a guide not exceed 2 ml/kg/h. 0.9% “normal” saline has often been the fluid of choice for this purpose; however, concerns about both the sodium and chloride load may favor other “balanced” fluids [13, 50, 51]. This fluid can again be substituted for the one designed specifically for maintenance of daily fluid and electrolyte requirements once certainty regarding fluid requirements and responsiveness has been reached. During this period, the solutions infused as medications may also be made more concentrated to limit volume requirements.

It must be remembered that intravenous fluids are drugs providing both electrolytes and water. While calculating these requirements, the patient’s medication and feed also need to be incorporated to avoid excessive volumes of each.

### Assessing volume status

#### Fluid responsiveness

Only half of the ICU patients with hemodynamic instability are able to “respond” to fluid loading, which is explained by the shape of the Frank–Starling curve [52]. On the initial and steep limb of the curve, the stroke volume is highly dependent on preload: Administering fluid will actually result in a significant increase in stroke volume. In contrast, if the heart is working on the terminal and flat portion of the Frank–Starling curve, it cannot utilize any preload reserve and fluid administration will not significantly increase stroke volume. Accordingly, predictors of volume responsiveness are mandatory to distinguish between patients who can benefit from fluid and those in whom fluid is useless and hence deleterious.

1. Static markers of cardiac preload

Considering the Frank–Starling relationship, the response to volume infusion is more likely to occur when the ventricular preload is low, rather than when it is high. Unfortunately, none of the measures of cardiac preload enables to accurately predict fluid responsiveness: Neither the central venous pressure (CVP), the pulmonary artery occlusion pressure (PAOP), nor the left ventricular end-diastolic area (LVEDA) can discriminate between responders and non-responders to fluid therapy [24, 53, 54]. Only the right ventricular and the global end-diastolic volume have been proven to be of some benefit compared to barometric preload indicators especially in patients with increased intra-thoracic or intra-abdominal pressures [23, 55].

2. Dynamic markers of volume responsiveness

The alternative method for predicting volume responsiveness is simply to induce a change in cardiac preload and to observe the resulting effects on stroke volume or cardiac output or any available surrogate, i.e., to perform a “functional assessment” of the cardiac function [24]. This is achieved with intravenous fluid boluses [56]. This method can be criticized because repeated infusions of such amounts could eventually exert adverse effects if there is no preload reserve, especially if pulmonary permeability was increased.

#### The respiratory variation of hemodynamic signals

Observing the respiratory variation of hemodynamic signals has emerged as an alternative for assessing volume responsiveness without administering fluid. The concept is based on the assumption that the cyclic changes in right ventricular preload induced by mechanical ventilation should result in greater cyclic changes in left ventricular stroke volume when both ventricles operate on the steep rather than on the flat portion of the Frank–Starling curve, i.e., in case of biventricular preload preserve.

Numerous studies have consistently demonstrated that the magnitude of respiratory variation of surrogates of stroke volume allows predicting fluid responsiveness with accuracy [24]. Pulse pressure variation (PPV) is the most popular index, since it needs only an arterial catheter to be obtained and numerous bedside monitors calculate and display its value in real time. Reliability of PPV to predict fluid responsiveness has been demonstrated in ICU patients when it is calculated from a simple arterial catheter [4] or automatically calculated by simple bedside monitors such as the IntelliVue (Philips, USA) [5], the PiCCO (PULSION Medical Systems SE, Germany) [6] and the LiDCOplus (LiDCO Group PLC, UK) [7] monitors [57–61]. PPV can also be automatically obtained with the LiDCOrapid (LiDCO Group PLC, UK), Mostcare (Vytech, Italy) and Pulsioflex (MAQUET, Germany) uncalibrated monitors [62]. Noninvasive finger pressure monitors such as the CNAP (CNSystems Medizintechnik AG, Austria) or ClearSight (Edwards Lifesciences Corporation, USA) also allow calculation of noninvasive PPV [63, 64]. It must be noted that this modality is difficult to use in several cases of trauma resuscitation as it requires controlled ventilation (i.e., no spontaneous ventilatory efforts), regular sinus rhythm and tidal volumes of >7 ml/kg.

#### Other markers

The following other surrogates of stroke volume respiratory variation can be used at the bedside:

- Respiratory variation of the pulse contour-derived stroke volume measured by the PiCCO or by the FloTrac/Vigileo (Edwards Lifesciences Corporation, USA) or by the LiDCOplus [58, 65, 66].
- Respiratory variation of the subaortic flow assessed by echocardiography and respiratory variation of the descending aortic blood flow assessed by esophageal Doppler [30, 67, 68].
- Other heart–lung interaction indices like respiratory variation of inferior or superior vena cava diameter (echocardiography), although limitations exist [24, 68–70].
- The passive leg raise (PLR) test carries an excellent ability to serve as a test of preload responsiveness, demonstrated in patients with acute circulatory failure [71, 72]. A 10–12% increase in cardiac output or stroke volume during PLR enables prediction of fluid responsiveness, even patients with cardiac arrhythmias and/or spontaneous ventilator triggering [73]. However, in conditions of increased IAP and pain, the PLR may result in a false negative [55, 74].
- The end-expiratory occlusion test. The end-expiratory occlusion test can be used in patients with low lung compliance [64, 75].
- Ultrasonography: This bedside modality has advantages of repeatability, being noninvasive, with the ability to assess dynamic changes in the inferior vena cava (IVC) diameter, left ventricular outflow tract stroke volume variation, and estimate cardiac ejection fraction [76]. This provides the ability for real-time guidance of fluid resuscitation [77]. The most widely used method for assessing fluid responsiveness using IVC parameters is the caval index [78]. This measurement is most useful at extremes of volume status and is influenced by increases in ventilation parameters (tidal volume and positive end-expiratory pressure) and intra-abdominal pressure. As a result, despite positive findings in early studies, research has demonstrated a limited ability to detect those patients that would respond to further fluid resuscitation due to changes in these ventilatory parameters, and other patient factors such as obesity [79, 80]. Other measurements using ultrasonography are possible such as SVV using pulse-wave Doppler, and aortic blood flow velocity using trans-esophageal echocardiography, but require more experience and may prove challenging in the emergency setting [81–83]. The combination of using ultrasonography to measure aortic velocity–time integral and combining this with a PLR test may be the best technique in skilled hands [84, 85].

Recommendations for current best practices:

1. If ultrasonography is available, then we advise using dynamic changes in IVC together with other clinical parameters. Preferably, if the skills are available, combining aortic velocity–time integral with dynamic changes from a PLR test should be performed.
2. In the absence of ultrasonography, dynamic markers of volume resuscitation should be attempted such as a PLR test, although this may have limited utility in the emergency setting due to patient injuries and pain.
3. If it is unable to utilize ultrasonography or PLR testing, other dynamic markers of fluid responsiveness can be attempted although these may be impractical in the emergency setting. Repeated “mini-boluses” of intravenous fluid (100–250 ml) can be used, provided that vigilance regarding excessive or inappropriate clear fluid administration is maintained.

## Special groups

### Pediatrics

Infants and children suffer from trauma, particularly vehicular trauma, with an increasing incidence in the developing world [86]. Children differ from adults in having a larger circulating blood volume (80 ml/kg in a term neonate) that decreases with age to the adult level of 70 ml/kg [87]. While the relative blood volume is larger, the absolute volumes required are small and should ideally be delivered through flow control devices (infusion pumps/syringe drivers) [88]. A cheap precaution is to deliver all clear fluids through a 60-drop-per-minute administration set rather than the 10-/15-drop-per-minute sets used for adults.

Restoration of circulating volume is a priority with the establishment of vascular access via a peripheral line, central (commonly femoral) line or intra-osseous line. The access should not be established distal to a site of injury (e.g., femoral line with blunt abdominal trauma) as the resuscitation fluid will extravasate into the injured area [89].

The principles of goal-directed therapy apply equally well to children as to adults. Initial resuscitation should be with 20 ml/kg of balanced crystalloid [90]. There is limited information on the efficacy and safety of synthetic colloids (e.g., HES) in children, with some evidence that hemodynamic goals are achieved more quickly and with smaller volumes but at increased cost and with no evidence of outcome benefit [91].

The role of all clear fluids is limited in trauma resuscitation due to their adverse effects of dilutional coagulopathy and anemia and generation of edema that hinders tissue perfusion and promotes organ dysfunction (including ileus, abdominal compartment syndrome and ARDS). The volume of clear fluid should not exceed 40 ml/kg [90]. Administration of blood and blood products (platelets and plasma) should be considered depending on the response to the initial 20 ml/kg crystalloid bolus and the severity of injury. Due to the small volumes required, many pediatricians use human colloids such as plasma or albumin for intravascular volume replacement in preference to synthetic clear fluids [92].

During the maintenance phase of resuscitation, children appear to be at risk of hyponatremia. This seems to be due to administration of excessive volumes of hypotonic solutions such as ½ strength Darrow’s solution (Na-61, K-12, Cl-52, Lactate-27 mmol/l) with 5% dextrose resulting in hyponatremia and potentially fatal cerebral edema [93]. A clear distinction is required between resuscitation fluids, that must be isotonic and preferably balanced, and maintenance fluids, that may be hypotonic and should only be given in limited volumes (maximum 2 ml/kg/h) via a flow controller to prevent rapid administration [94].

### Elderly

The World Health Organization defines “elderly” as a chronological age of 65 years or more. Age is only one criterion in the assessment of overall health leading to the concept of biological age based on organ dysfunction and/or chronic disease [95]. Despite advances in trauma care, the elderly, either chronologically or biologically, are at increased risk of morbidity, particularly limitation of mobility and self-care ability, and mortality after trauma [96].

Cardiovascular changes of aging include stiffening of the arterial circulation and loss of compliance of the left ventricle. The elderly thus tolerate hypo- and hypervolemia poorly. Volume loss reduces preload resulting in ventricular under-filling and a disproportionate drop in cardiac output. Over-hydration is as dangerous due to the lack of ventricular compliance predisposing to the development of edema, particularly pulmonary edema [97]. Assessment of fluid requirements in the elderly is best done by echocardiography as noninvasive measurements based on pressure or pulse contour analysis are subject to variation due to the changes in the cardiovascular system from aging [98].

Clear fluid administration should initially be limited to 20 ml/kg with early consideration given to the administration of blood and blood products. Careful re-evaluation and vigilant monitoring should to performed to determine if further fluid administration is required; particularly if underlying heart disease is suspected. Due to the likelihood of underlying coronary and cerebral artery disease in the elderly, consideration should be given to maintaining hemoglobin levels above 9 g/dl and mean arterial pressure above 70 mmHg, particularly if the patients comorbidities are unknown [97].

### Burns

Appropriate and effective initial resuscitation of victims of burns is vital for survival and reduction of morbidity and mortality [99]. The deeper and more extensive the burn, the greater the fluid requirements, but excessive fluid administration will also increase morbidity by generation of edema [100]. The formulas used for calculating volume requirements (e.g., Brooke & Parkland) use only body surface area (BSA) and do not compensate for depth. In clinical practice, the fluid requirement is approximately 5 ml/kg/ %BSA during the first 24 h [101].

The rate of fluid administration will initially be rapid with up to half the daily requirement given in the first 6 h. The use of colloid solutions is controversial. Hyperoncotic colloids worsen outcome, but the role of albumin or synthetic colloids (e.g., HES) is less clear [102–104]. Colloid solutions shorten the time to achieve hemodynamic goals, but increase expense without a concomitant improvement in outcome.

Enteral resuscitation is effective if commenced within 6 h. Placement of a feeding tube should be part of the resuscitation protocol for burns. Delay of feeding for more than 6 h will result in an increasing feeding failure due to gastroparesis and ileus. Maintenance of enteral feeding maintains the gut associated lymphoid tissue that participates in maintenance of immunity at all epithelial surfaces including the skin. A standard formula may be used starting at 2 ml/kg/h and increasing incrementally every 3 h until the goal rate calculated for each patient is reached [105].

Two simple investigations should be used to monitor the effectiveness of resuscitation from burns. The first is the hematocrit, which may be as high as 70% on admission after extensive burns. Failure to reduce the hematocrit below 40% within the first 6 h is an accurate indicator of poor prognosis. The second is urine output, which should be maintained at around 1 ml/kg/h. Development of acute renal failure carries a very poor prognosis with extensive burns [106]. A more detailed review of fluids in burn resuscitation is beyond the scope of this paper; however, further references are provided [107, 108].

### Crush injury/syndrome

Crush injury is seen in victims of motor vehicle collisions who are entrapped and have limbs compressed, resulting in direct muscle trauma followed by a reperfusion injury when freed. Similar injury is seen in prolonged immobilization (after a fall or drug overdose) and entrapment in collapsed buildings after natural disasters [109]. South Africa has an unfortunate history of interpersonal violence. With the breakdown in the rule of law in many communities, alleged criminals may be assaulted by community members using traditional whips (sjamboks). This results in extensive muscle injury; however, muscle perfusion is maintained so reperfusion does not occur [110]. Muscle injury releases myoglobin that is detrimental to kidneys. A surrogate marker for myoglobin is creatine kinase (CK) used as follows (Table 3).

**Table 3:** Representation of how to use creatine kinase as a surrogate marker for myoglobin

CK U/l	Risk of renal failure	Admission
<500	Low	Unlikely
500–5000	Intermediate	At least overnight
>5000U/l	High	Admission to ICU/high care

With diffuse injury, such as community sjambok assaults, the surface area of the body injured should be quantified as for burns. A surface area of >18% carries increased risk of renal dysfunction [110]. Aggressive fluid loading (20–40 ml/kg initial bolus followed by 10–20 ml/kg/h) should begin as soon as the patient makes contact with the healthcare system. In the pre-hospital environment, fluid loading should ideally occur prior to release of crushed limb/s [111].

Traditionally, 0.9% saline is used for fluid loading. Alternatives, to limit the occurrence of hypernatremia and hyperchloremic acidosis, include 0.45% saline and alternating 0.9% saline and 5% dextrose. Balanced solutions such as modified Ringer’s lactate are not recommended due to concerns regarding hyperkalemia, but this risk may be offset by the hyperchloremic acidosis seen with large-volume saline administration [109].

Should presentation be delayed, creatinine and potassium should be measured while initial fluid loading occurs, as the kidneys may have been damaged beyond immediate recovery. Failure to produce urine after initial fluid loading associated with an elevated urea, creatinine and potassium indicates the need for urgent renal replacement therapy. Further fluid loading should not be administered, as the absolute volume overload that arises in the absence of urine output will result in pulmonary edema with hypoxia requiring intubation and ventilation. There is no role for loop or osmotic diuretics, and the use of sodium bicarbonate to induce alkaline diuresis is also not supported by evidence [110].

### Pregnancy

Trauma, particularly vehicular, or due to intimate partner violence is a common cause for maternal and fetal morbidity and mortality. In the developing world, neonatal intensive-care facilities are limited so maternal considerations take precedence in resuscitation until fetal viability is likely [112].

Pregnancy duration of more than 20 weeks makes aortocaval compression a realistic cause of hypotension during resuscitation, emphasizing the need for maintaining a 20° left lateral tilt [113]. Fluid administration follows accepted principles of resuscitation [112].

Should delivery occur during resuscitation, significant blood loss may occur due to post-partum hemorrhage. Oxytocin availability may be limited due to expense and requirement for refrigeration. Misoprostol is an accepted alternative, but is only available in an oral form that may need to be administered rectally during resuscitation [114]. It should be remembered that the physiological compensation for blood loss might be better tolerated in pregnancy due to the physiological changes that predominantly take place in the second and third trimesters and include an increased circulating blood volume and cardiac output. Awareness of this should be maintained to avoid underestimation of blood loss and underlying injuries.

## Conclusion

Fluids are drugs and should be managed as such. Appropriate early fluid resuscitation in trauma patients is a challenging task. Care should be taken in selecting both the type and volume to promote appropriate perfusion and oxygen delivery, avoiding the adverse effects seen when giving too little or too much. Ongoing fluid strategies following resuscitation should incorporate dynamic markers of volume status whenever possible. All aspects of fluid administration should be incorporated into daily fluid plans, including feeding and infusions of medications. A sound knowledge of the differences and physiological consequences of specific trauma groups is essential for all practitioners delivering care for trauma patients [7].
